# Molecular Diagnosis and Genetic Counseling of Cystic Fibrosis and Related Disorders: New Challenges

**DOI:** 10.3390/genes11060619

**Published:** 2020-06-04

**Authors:** Thierry Bienvenu, Maureen Lopez, Emmanuelle Girodon

**Affiliations:** Molecular Genetics Laboratory, Cochin Hospital, APHP.Centre–Université de Paris, 75014 Paris, France; thierry.bienvenu@inserm.fr (T.B.); maureen.lopez@aphp.fr (M.L.)

**Keywords:** cystic fibrosis, CFTR, CFTR-related disorders, molecular diagnosis, CFTR variants, Next Generation Sequencing (NGS), disease liability, interpretation, penetrance, genotype-guided therapy

## Abstract

Identification of the cystic fibrosis transmembrane conductance regulator *(CFTR)* gene and its numerous variants opened the way to fantastic breakthroughs in diagnosis, research and treatment of cystic fibrosis (CF). The current and future challenges of molecular diagnosis of CF and CFTR-related disorders and of genetic counseling are here reviewed. Technological advances have enabled to make a diagnosis of CF with a sensitivity of 99% by using next generation sequencing in a single step. The detection of heretofore unidentified variants and ethnic-specific variants remains challenging, especially for newborn screening (NBS), CF carrier testing and genotype-guided therapy. Among the criteria for assessing the impact of variants, population genetics data are insufficiently taken into account and the penetrance of CF associated with *CFTR* variants remains poorly known. The huge diversity of diagnostic and genetic counseling indications for *CFTR* studies makes assessment of variant disease-liability critical. This is especially discussed in the perspective of wide genome analyses for NBS and CF carrier screening in the general population, as future challenges.

## 1. Introduction

Cystic fibrosis (CF) is one of the most frequent life-limiting autosomal recessive diseases, characterized in its classical form by chronic pulmonary obstruction and infections, pancreatic insufficiency, male infertility, sweat chloride concentrations ≥60 mmol/L and two loss-of-function variants in the cystic fibrosis transmembrane conductance regulator (*CFTR*) gene (NM_000492.4; LRG_663t1) [1]. With the implementation of newborn screening (NBS) for CF, an increasing number of diagnoses are now made in asymptomatic patients. *CFTR* variants have diverse effects on expression and function of the CFTR protein, which principally acts as an ATP-gated chloride channel. Its absence or dysfunction leads to ion flux perturbations in the epithelial cells of various organs involved in CF [2].

Since the discovery of the *CFTR* gene more than thirty years ago, considerable scientific advances have made CF a model in terms of comprehensive knowledge of a genetic disease, molecular diagnosis, genetic counseling and personalized medicine. Technical milestones have led to identify a huge number of *CFTR* gene variants and a variety of molecular mechanisms responsible for CF [3], which contribute to the wide phenotype variability, and to achieve one of the highest sensitivities in the diagnosis of a hereditary disease, more than 99% of CF-causing variants being identified in newborns with CF [4]. The advent of next generation sequencing (NGS)-based technologies has deeply modified laboratory’s practice, improved genotyping coverage and questioned screening policy. Specific molecular tools have also been developed for preimplantation genetic diagnosis [5] and non-invasive prenatal diagnosis of CF [6]. Other clinical entities related to *CFTR* variants have been described since the early 90s, with a continuum of CFTR dysfunction, from CFTR-related disorders (CFTR-RD), which are defined by evidence of CFTR dysfunction but do not meet the criteria for a CF diagnosis [7], to a number of conditions associated with a higher proportion of CF carriers compared to the general population, such as asthma or bronchopulmonary allergic aspergillosis [8]. Very recently, Miller et al. reported an increased risk for 57 CF-related symptoms in CF carriers in a study that questions the relevance of detecting CF carriers for preventive care [9]. CFTR-RDs principally include isolated male infertility by congenital bilateral absence of the vas deferens, idiopathic pancreatitis, disseminated bronchiectasis and chronic rhinosinusitis. The contribution of *CFTR* variants however varies from one condition to another, and may act in a multifactorial context, with other genes being potentially involved, such as *ADGRG2* in male infertility by the absence of vas deferens [10] or *PRSS1*, *SPINK1* and *CTRC* in pancreatitis [11]. There is thus a huge diversity of diagnostic and genetic counseling indications for searching *CFTR* variants, and appropriate tools should be used to answer clinical questions [1,12,13]. A great challenge in 2020 remains to accurately assess the disease liability of *CFTR* variants in the appropriate clinical context and to determine whether a variant should be reported as disease-causing, whether a genotype is compatible with the phenotype or which phenotype is compatible with the genotype identified in the context of NBS for CF, and whether a variant should be considered for genetic counseling purposes. The availability of genotype-guided therapy has also brought a renewed interest in deciphering the impact of *CFTR* variants.

In the present article, we review the current and future challenges of molecular diagnosis and genetic counseling. Despite a very high sensitivity of molecular tools, characterization of heretofore unidentified disease-causing variants in patients remains a technical challenge. We will also focus on ethnic-specific variants, the detection of which being challenging for NBS, CF carrier testing and genotype-guided therapy. We will then emphasize on the importance to consider population genetics and penetrance data in the process to evaluate the impact of variants. These data will eventually be discussed in the perspective of implementation of wide genome analyses for NBS and preconception CF carrier screening in the general population in an increasing number of countries.

## 2. Molecular Diagnosis

The following section reviews the process to achieve a molecular diagnosis of CF or CFTR-RD, by using a panel of tools in successive stages and also covers the indications of prenatal diagnosis and carrier testing.

### 2.1. Molecular Diagnosis of CF and CFTR-RD

A CF diagnosis is suggested by characteristic symptoms, a family history of CF (most often in a sibling) or a positive CF NBS result and is confirmed by evidence of CFTR dysfunction, most often by abnormal sweat chloride test results or by identification of two CF-causing alleles, one on each parental gene [1]. Diagnostic algorithms also include other CFTR functional investigations such as trans-epithelial nasal potential difference measurement or intestinal current measurement on rectal biopsies [1]. The diagnosis of CFTR-RD is established in the event of symptoms suggestive for CFTR dysfunction but when the biological criteria for CF are not met. These clinical entities essentially include monosymptomatic disorders in adults but the distinction between a mild CF and a CFTR-RD with multiorgan involvement may be artificial. Despite limitations, as for all tests, and the number of inconclusive situations, *CFTR* genetic analysis is a cornerstone for the diagnosis of CF and CFTR-RD. In adult patients having a monosymptomatic disease suggestive of a CFTR-RD, the proportion of homozygotes or compound heterozygotes is lower than in CF patients. It was shown to be as high as 82% in male infertility by absence of vas deferens [14] but low in other entities [7], e.g., 6.3% in aquagenic palmoplantar keratoderma [15]. Diagnostic sensitivities also vary between studies, depending on inclusion criteria and investigations for other causes, such as for disseminated bronchiectasis or pancreatitis. Provision of clinical and biological information prior to *CFTR* testing is thus essential to ensure that appropriate studies are carried out and that accurate interpretation is given.

More than 2000 *CFTR* variants have been reported to the Cystic Fibrosis Mutation Database [16], identified in patients with CF, a CFTR-RD or various other clinical presentations and in healthy individuals. Beside frequent variants that account for 50%–90% CF alleles worldwide, the majority of *CFTR* variants are as rare as to be called “private”, as they are only present in individual families, or could be specific of an ethnic population. *CFTR* variants have been classified into five categories according to their clinical consequence: CF-causing variants, which are responsible for CF when combined in *trans* with a known CF-causing variant; CFTR-RD-causing variants, which are observed in patients with a CFTR-RD when combined in *trans* with a CF-causing variant; variants of varying clinical consequences (VVCC), which are reported as well in CF patients as in patients with a CFTR-RD when in *trans* with a CF-causing variant; variants of unproven or uncertain clinical significance (VUS) and variants with no clinical consequences [12]. Molecular diagnosis of CF and CFTR-RD is thus challenging especially due to the high heterogeneity of variants and genotypes and the difficulty to accurately evaluate their impact.

### 2.2. Tools and Strategies Used for the Molecular Diagnosis of CF and CFTR-RD

**Figure 1:**
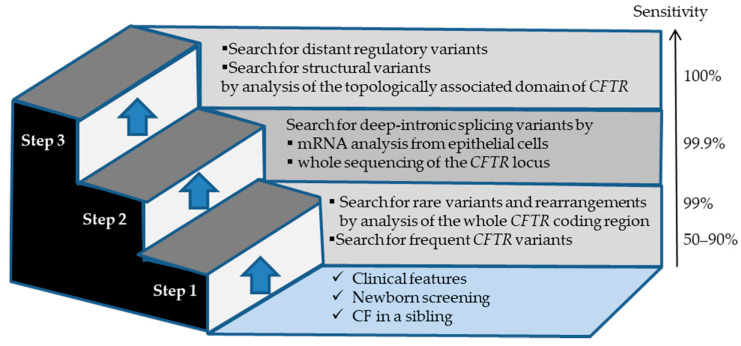
Molecular investigation for the diagnosis of cystic fibrosis (CF) and cystic fibrosis transmembrane conductance regulator related disorders (CFTR-RD) in three steps. Sensitivity refers to variant detection rate in patients with CF.

Robust strategies and cutting-edge methods have been steadily developed to identify *CFTR* variants, to study their impact and to predict their pathogenicity. A diagnosis may be achieved in three successive molecular steps, the implementation of which depending on the results of each previous step (Figure 1). The first step still often starts with the detection of the most frequent disease-causing variants using different commercially available kits, very often CE-marked for in vitro diagnosis. The sensitivity of variant panels greatly varies according to geographic/ethnic origins. For example, the sensitivity (or variant detection rate) of the Elucigene^®^ CF-EU2v1 kit (Elucigene^®^, Delta Diagnostics, Manchester, UK) targeting 51 *CFTR* variants, varies from 93% in Ireland [17] to 49% in Turkey [18]. Whenever necessary, according to various strategies depending on the clinical situations and to national algorithms for CF NBS, which mostly include a prior step of sweat testing, rare variants are then searched by Sanger sequencing or NGS analysis of the 27 coding regions of the *CFTR* gene, targeted intronic regions containing known deep-intronic disease-causing variants, and part of the promoter. The NGS-based approach enables the simultaneous detection of single nucleotide variants and large deletions or duplications encompassing one or several exons [19]. For practical, organizational and economic reasons, some laboratories have now applied NGS as the single technique in their routine practice, possibly in two steps, as implemented in a few CF NBS programs, notably considering multi-ethnic populations [20]. Variant detection rate of this comprehensive step proved to be as high as 99% in CF newborns [4].

The second step concerns about 2% of patients with a high clinical suspicion of CF who carry only one CF-causing variant, and theoretically 0.01% of CF patients who carry no CF-causing variant. It is focused on the identification of rare or unknown deep-intronic variants, which may affect the structure, the size and/or the sequence of the *CFTR* messenger RNA (mRNA). This can be done either by studying *CFTR* mRNA of patients’ epithelial cells, which are easily obtained by nasal brushing [21,22], or by analyzing the whole *CFTR* locus by NGS [23,24]. The disadvantages of mRNA studies are the requirement of another sample from a specific tissue and the instability of abnormal transcripts containing stop codons, which may thus be hardly or not detected. The limitations of sequencing the whole gene are the cost to analyze the large-sized introns and the complexity to evaluate the impact of numerous identified variants [23].

After the second step, very few patients with CF still have an incomplete genotype. The third step, which is not performed in a clinical setting yet, aims to search for variants in the distant regulatory elements that may quantitatively alter CFTR expression [25], as well as large structural variants (such as duplications, deletions, inversions and translocations of blocks of DNA sequence). This can be achieved by resequencing a large genomic region including the entire topologically associated domain of *CFTR* on NGS specific platforms.

### 2.3. Prenatal and Preimplantation Diagnoses of CF

When applied to couples at-risk of ¼ or ½ of having a child with CF, the molecular strategy applied for prenatal diagnosis is simpler and consists of the detection of the known CF-causing variants that were previously identified in the index case or the parents. Considerable progress has been made in the field of preimplantation genetic diagnosis [5] and non-invasive prenatal diagnosis. Early detection of paternal CF variants in maternal blood has been routinely available for a few years [6,26] and, in the very near future, non-invasive procedures should be available to all at-risk couples through NGS haplotype-based approaches [27].

Prenatal diagnosis of CF may also be performed in the absence of family history of CF if ultrasound digestive abnormalities such as fetal echogenic bowel, fetal intestinal loop dilatation and non-visualization of the fetal gallbladder are observed during pregnancy [28]. Depending on national regulations, the term of pregnancy and ultrasound signs, the strategy followed and extent of the study may be the same as for diagnosis. Ensuring coverage of population-specific variants in this context is critical.

### 2.4. Recommendations for Population-Based CF Carrier Screening

Identification of CF-causing variants among all *CFTR* variants is of utmost importance, as only they are considered for CF carrier testing and prenatal diagnosis with subsequent termination of pregnancy. In 2002, the American College of Medical Genetics defined guidelines for clinical genetics laboratories [29]. Although over 900 variants were already described, a minimum variant panel for population-based carrier screening purposes was defined, consisting of 25 variants, restricted to 23 two years later, based on a frequency above 0.1% in CF chromosomes in a pan-ethnic population [30] (Table S1). These recommendations were needed to focus on true CF-causing variants and establish a rapid CF carrier diagnosis but presented limitations for specific ethnics groups, so that variant panels had to be tailored accordingly and many of them were considerably expanded. In order to document the highly variable variant distribution and frequency among populations, a systematic search in PubMed was made using keywords “CFTR”, “cystic fibrosis”, “variant” or “CFTR”, “cystic fibrosis” and “mutation”. Recent data in specific ethnic populations for which little was known were chosen to illustrate the variable representativeness of the 23 variant panel, with cumulated frequencies varying from 3% to 91% depending on the ethnic groups (Table S1). Moreover, in some of them, the majority of CF patients carried at least one population-specific variant, such as c.3276C>G (Y1092X) in Cameroon, c.3310G>T (E1104X) in Tunisia [31] or c.3700A>G (I1234V) in Qatar and in certain Arab tribes [32]. Although ethnicity-based genetic testing may appear obsolete with the wide implementation of NGS, the challenge remains to ensure the coverage of population-specific CF-causing variants wherever appropriate, especially as the NGS-based approach is not affordable in all countries.

### 2.5. Impact of Variant Heterogeneity on Personalized Medicine

Development of drugs to correct, enhance and stabilize the CFTR protein has made *CFTR* genotyping crucial to optimize therapy. It has renewed interest for the original variant classification that was based on functional data, six classes being recognized according to the CFTR protein defect in: I. synthesis, II. processing and maturation, III. gating, IV. conductance, V. abundance due to reduced amount of normal mRNA and VI. stability at the membrane [33,34,35]. However, clinical trials have showed that numerous variants caused pleiotropic defects, such as the most frequent CF-causing variant worldwide, c.1521_1523del (F508del) [36], thus justifying the use of drug combinations. Ivacaftor was the first drug to be US Food and Drug Administration approved for the treatment of CF, efficiently targeting the gating defect of class III variants, and it was further extended to class IV variants (Table S2). The list may however differ with that approved by the European Medicines Agency. These variants represent only 2%–10% of CF-causing variants in several populations and are totally absent in other ethnic groups (Table S2). Moreover, the clinical significance of several of them is questionable. Since then, combinations of ivacaftor with other molecules that aim to increase CFTR protein trafficking to the plasma membrane have been approved for specific variants: lumacaftor/ivacaftor in F508del homozygous patients; tezacaftor/ivacaftor in patients carrying F508del and a variant associated with residual CFTR function and elexacaftor/tezacaftor/ivacaftor in patients carrying at least one copy of F508del. The monumental interest of the triple-combination is that a large proportion of patients with CF are eligible for treatment in numerous populations. However, in specific ethnic groups, F508del is absent (such as Iraq, Sudan, Qatar, Japan and China) or extremely rare (Jordan 6%–7%, Bahrain 8%) [37] (Table S1). The challenge is thus to make personalized therapy accessible to all patients. Other strategies are still under development, such as amplifiers that increase the amount of CFTR available for modulators, readthrough agents targeting in frame premature termination codons and gene therapy [38].

## 3. How to Assess the Impact of *CFTR* Variants—The Challenge of Penetrance

**Figure 2:**
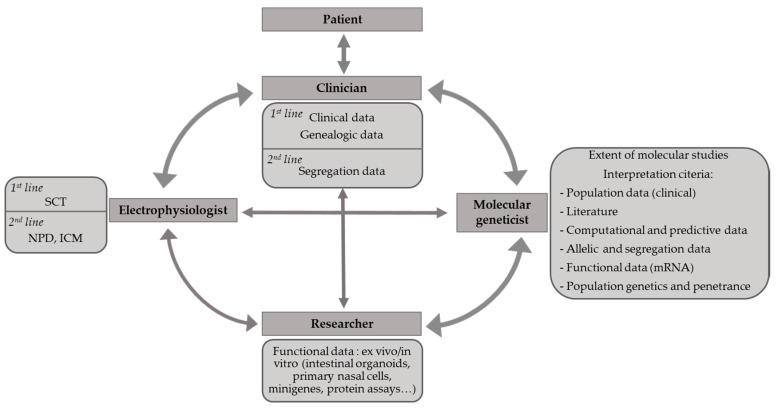
Links between health care professionals for carrying out appropriate *CFTR* studies and accurate interpretation of *CFTR* genetic test results. NPD: nasal potential difference; ICM: intestinal chloride measurement; SCT: sweat chloride testing.

Assessing the impact of *CFTR* variants is a comprehensive process, which allows one to answer clinical questions that cover diagnostic, genetic counseling and therapeutic issues. It is a daily challenge in specialized CF molecular laboratories, especially when dealing with rare or unknown variants. It relies on a good dialogue between clinicians, electrophysiologists and molecular geneticists, with appropriate clinical information provided by the clinicians, and occasionally requires the expertise of researchers (Figure 2). In 2015, international recommendations for interpretation and classification of sequence variations were published [39], based on diverse criteria including population, computational, segregation and functional data. They are reviewed in the present section. The recommended phenotypic classification into five categories, that is, “pathogenic”, “likely pathogenic”, “uncertain significance”, “likely benign” and “benign” is however not completely equivalent as that used for *CFTR* variants and does not take into account phenotypic diversity, as “pathogenic” may be used for CF-causing and CFTR-RD-causing variants. As an example, variant c.1210-34TG[12]T[5] (TG12T5) may be considered pathogenic in the context of male infertility by absence of vas deferens but not in the context of CF.

### 3.1. Population Data (Clinical)

Assessing the impact and potential clinical consequence of a *CFTR* variant starts with search for clinical observations related to this variant in laboratory’s own database, publicly available databases and the literature. Considering the class of variants in *trans* is essential in the context of a recessive disorder as CF. Beside the original CF Mutation Database, which most often describes the original observation and provides links to literature in PubMed, two other locus specific databases provide substantial and complementary information on variants and also provide links to epidemiological, computational, functional and literature data (Table 1). CFTR2 collects data from North American and European national CF patient registries [40], i.e., only from patients diagnosed with CF. *CFTR*-France is dedicated to the interpretation of rare variants and is built on data from genetics laboratories and the French CF patient registry [41]. It collects genetic and clinical data from patients with CF and CFTR-RD and from asymptomatic individuals who are compound heterozygous for two *CFTR* variants. Due to different designs, these two databases may provide discordant information and variant classification one should be aware of. In particular, the class of CFTR-RD-causing variants is not referenced in CFTR2 and variants classified as CFTR-RD in *CFTR*-France may be found either as VVCC or not CF-causing in CFTR2. Other general databases such as ClinVar [42], and Human Gene Mutation Database^®^ [43], also provide clinical information and variant classification, mostly based on literature data, but numerous variants have been overclassified as “pathogenic” [44].

### 3.2. Literature Data

The literature review may provide valuable information for clinical, population genetics and functional data.

### 3.3. Computational and Predictive Data

In silico bioinformatics tools help predict the impact of variants at the mRNA level or the protein level, based on conservation of amino acids within different proteins of the same family, evolutionary conservation within species and biochemical distance between amino acids. Importantly, any intronic or exonic variant, even synonymous, may alter splicing. There are some limitations with using in silico tools, notably because of challenges in prioritizing one tool over the other and the lack of reliable standard interpretation guidelines [19]. Cystic fibrosis missense analysis (CYSMA) is a recently developed website dedicated to *CFTR* missense variants based on integrated in-house bioinformatics tools, which proved efficient to predict the impact of *CFTR* variants [46].

With the advent of wide genome analysis, aggregators that combine multiple evaluation tools have been implemented, such as Varsome [47] or Intervar [48]. They may be of help but exhibit significant limitations for *CFTR* variants, displaying a high degree of uncertainty for numerous variants [44].

### 3.4. Allelic and Segregation Data

Genetic studies in the parents of a patient are necessary to confirm the status of homozygous for a variant or compound heterozygous for two variants. Inheritance of two variants from the same parent (in *cis*) indicates the presence of a complex allele and numerous frequent or rare complex alleles have been described in the *CFTR* gene [41,49,50]. Complex alleles increase the complexity of *CFTR* variant classification, as illustrated for the c.445G>A (G149R) CF-causing variant and the c.1327G>T (D443Y) CFTR-RD-causing variant, each of them being described in *cis* with an already frequent complex allele c.[1727G>C;2002C>T] (G576A;R668C) [51]. *Cis*-variants may also affect the response to CFTR modulators, which impacts on reporting genetic test results [52]. De novo occurrence of variants is extremely rare in CF but has been described [53], also justifying parents’ study. Abnormal segregation during parents’ study may also unmask the presence of a deletion that would have escaped detection, depending on the techniques used [54].

### 3.5. Functional Data

Evidence of CFTR dysfunction may be brought by different categories of investigation, including in vivo, ex vivo and in vitro tests. While sweat testing is most often performed before comprehensive genetic testing in children, it may be performed after identification of *CFTR* variants in newborns, depending on NBS programs, or in adults with a possible CFTR-RD or CF. Nasal potential difference or intestinal current measurements are often used in the second line in case of inconclusive sweat test results or to help variant interpretation for patients carrying VUS. Likewise, other tests may be of help, such as sweat secretion after β-adrenergic stimulation, also called evaporimetry [55]. Ex vivo assessment of CFTR function on miniaturized versions of organs called organoids, from minimally invasive rectal biopsies [56] or on bronchial or nasal cells [57,58], is based on sophisticated and comprehensive techniques implemented in a few expert laboratories, which help diagnose, understand mechanistic defects and better predict organ-specific drug responses [59].

In vitro assays implemented to evaluate the impact of variants on *CFTR* mRNA or protein are also usually performed in a research setting. Minigene systems most often reproduce one or several exons in cloned plasmids, which are then transfected in human cells. They interrogate the impact of intronic or exonic variants on splicing [60,61] and are useful alternative tools when patients’ epithelial cells are not available for mRNA study. Importantly, they have allowed demonstration of a splicing impact of variants heretofore considered as missense, such c.2908G>C (G970R), which escapes CFTR modulator therapy [62] or c.3700A>G (I1234V) [63], or as nonsense, like c.2491G>T (E831X) [64]. Functional in vitro studies that focus on CFTR protein synthesis, maturation and function, are invaluable investigation tools. However, as noted above, many *CFTR* variants impair more than one single cellular process, as F508del [36]. Virtually no assay reflects the full biological function of the CFTR protein, so that the absence of defect observed does not rule out an impact on CFTR protein function. Eventually, numerous studies performed on presumed missense variants have also neglected a potential impact on splicing and should thus be considered cautiously.

### 3.6. Population Genetics and Penetrance Associated to *CFTR* Variants

Looking at variant frequencies in the general population has long been used to assess potential variant pathogenicity. A greater frequency of a variant in the ethnic-matched general population than in the population of CF patients, or greater than expected considering the frequency of CF, is a strong support for a benign interpretation. Data from the general population, globally and in specific population groups, are available on large reference datasets such as gnomAD [65]. Indeed, some variants are rarely found in our routine practice but are frequent in specific general populations, such as c.1666A>G (I556V), which allelic frequency in the Asian population reaches 4.7%, and c.2620-26A>G, which allelic frequency in the Ashkenazi Jewish population is 2.7%. These variants, which may have been overclassified in locus specific databases, should definitely be considered non-disease-causing. The occurrence of a rare or previously undescribed variant in *trans* of a known CF-causing variant in a healthy individual is also in favor of benignity.

**Figure 3:**
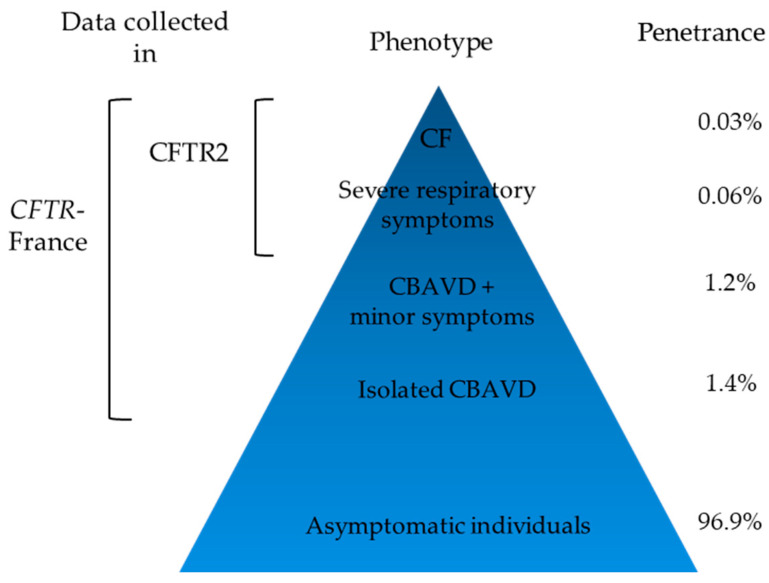
Penetrance of phenotypes in individuals who are compound heterozygous for c.[350G>A;1210-12T[7]];[1521_1523del] (R117H;T7/F508del) from Thauvin et al. [67].

Population genetics data proved useful to get an insight into the penetrance associated with *CFTR* variants. The penetrance of a phenotype is defined as the proportion of patients carrying a given genotype who develop this phenotype. For a recessive disease as CF, homozygous or compound heterozygous genotypes are most often detected in symptomatic patients and are described in clinical databases, which means that potential cases in healthy individuals are rarely taken into account, unless through family testing. CFTR2 thus represents the tip of the iceberg of all possible phenotypes associated with a variant. Few studies have shown an unexpectedly low penetrance associated with some *CFTR* variants, such as the c.1210-34TG[11]T[5] (TG11T5) variant [66], c.350G>A (R117H) [67] and other variants [40,68]. As an illustration, taking into account clinical observations and epidemiological data, a French collaborative study showed that the penetrance of CF in individuals compound heterozygous for R117H;T7 and F508del was as low as 0.03% and that of CFTR-RD was 3% [67] (Figure 3). Such comprehensive data are however not available for the huge amount of *CFTR* variants but incomplete penetrance may be supported by other lines of evidence. First, clinical observations and comparison of disease phenotypes in CFTR2 and *CFTR*-France databases suggest an incomplete penetrance of CF for variants that have been classified as CF-causing in CFTR2 but milder in *CFTR*-France, such as c.328G>C (D110H), c.349C>T (R117C) or c.617T>G (L206W). Second, the higher frequency of variants in the general population than in the population of CF patients is strongly against a severe deleterious effect, as for variants c.1210-12T[5] (T5), c.2991G>C (L997F) or R117H. Eventually, based on variant frequencies in the general population and results of the French NBS program over the 2002–2017 period, a recent study strongly suggested incomplete penetrance for 10 *CFTR* variants found in inconclusive cases after CF NBS [68]. The low penetrance associated with some variants such as the T5 variants might help clinicians to adapt medical care and follow-up of newborns carrying these variants, as well as genetic counseling given to families.

## 4. The Challenges of Genetic Counseling—When Genetic Counseling Meets Diagnosis

**Figure 4:**
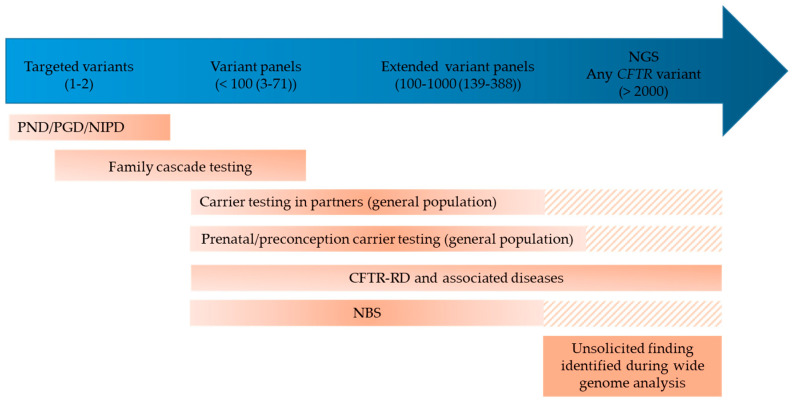
Genetic counseling situations (in orange), with potential identification of CF carriers, according to variable practices of molecular analysis (in blue). The number of variants tested is indicated in brackets. CFTR-RD: CFTR-related disorder; NBS: newborn screening; NIPD: non-invasive prenatal diagnosis; PND: prenatal diagnosis; PGD: preimplantation genetic diagnosis. Hatched lines: expected practice in the near future.

Genetic counseling for CF is a very important part of medical consultation and laboratory activities, which has become complex over the years (Figure 4). It has long been focused on the identification of CF carriers in the family of patients having CF and on provision of counseling services to couples in order to ensure informed reproductive decision-making [69]. Once a CF carrier is identified, testing for the most frequent CF-causing variants according to his/her geographic origins is recommended in the partner in a prenatal or preconception setting [13]. Extended *CFTR* sequencing analysis in the partners is however more and more performed, at least in partners of CF patients, because of the prior risk for the couple of having a child with CF (1/70, provided a CF carrier frequency of 1/35) [69]. Genetic counseling should also be considered for all symptomatic patients who carry *CFTR* variants, due to the potential risk of CF in the patients’ offspring and relatives. The identification of a CF variant leads to recommend CF carrier testing in the relatives and the partner in case of a parental project. While cascade testing for known CFTR-RD variants is not recommended, the identification of VVCC or VUS makes genetic counseling delicate and may be discussed on a case-by-case basis. Occasionally, *CFTR* testing in healthy adult siblings of a patient leads to identify the same genotype as in the patient. These individuals might develop symptoms related to a CFTR-RD or a mild form of CF, and thus need clinical investigation. On the other hand, such findings contribute to documenting the variable penetrance associated with some *CFTR* variants.

Identification of CF carriers may also occur in the absence of any family history of CF, during the process of NBS, or during preconception carrier screening or during wide genome analysis as an unsolicited or secondary finding (Figure 4). Expanded preconception carrier screening for CF and other recessive disorders is also under consideration in countries according to an overall positive attitude of the general population [70,71]. Preconception CF carrier screening has already been implemented in the US, Israël and Northeast Italy [72]. In most countries however, for practical reasons variant panels used for diagnostic purposes are also used for carrier testing. Again, it is important to discriminate true CF-causing variants from those that are CFTR-RD-causing, VVCC or non disease-causing. The wide implementation of NGS-based *CFTR* analysis in various clinical settings has increased this concern, with the identification of rare VUS or variants for which discrepant interpretation is found in databases or the literature. For genetic counseling purposes, the question of penetrance associated with variants is even more critical. Contrary to the diagnostic setting where the main question to answer is “does the genotype account for the phenotype?”, which may already be difficult with inconclusive genotypes, a challenging question in genetic counseling is to predict the phenotype resulting from the combination of a VUS or a VVCC with a known CF-causing variant.

Identification of carriers of CF-causing variants is also of particular concern in the context of CF NBS. On one hand, testing the parents may lead to identify another *CFTR* variant, possibly outside the NBS panel depending on laboratory’s practice. This result then raises the question of looking for this second variant in the infant who has a negative sweat test result. On the other hand, extended *CFTR* gene sequencing is being considered as part of the core strategy in an increasing number of programs [73,74]. Moreover, the relevance of implementing extended NBS for numerous genetic diseases is currently debated [74,75,76,77], also taking into account economical aspects. The introduction of NGS, with or without prior immunoreactive trypsinogen measurement and without filtering CF variants would lead to detect not only a higher number of carriers of known CF-causing variants but a much higher number of carriers of VVCC and VUS. The risk would be to consider neonates as carriers of a CF-causing variant and to offer inappropriate genetic counseling and testing in the family, and eventually inappropriate prenatal diagnosis.

The face of genetic counseling for CF will inevitably deeply change in the coming years. Health public policies of CF carrier screening in the general population aim to detect most CF carrier couples and prenatal requests may increase, especially with the availability of non-invasive procedures. This would ineluctably impact on the prevalence of CF births, which then would raise the question of the relevance of NBS if the incidence of CF is getting very low. In other respects, due to formidable progress in genotype-guided therapy, parents at risk of having a child with CF could prefer the option of continuation of pregnancy over that of termination. Prediction of changing attitudes and practices is a delicate business.

Very recently, a study conducted on 19,802 CF carriers who were matched each with five controls, reported a higher prevalence of 57 out of 59 CF-related symptoms or conditions in CF carriers than in the general population. These conditions included already known CFTR-RDs, conditions where a higher prevalence of *CFTR* variants has already been reported, such as allergic bronchopulmonary aspergillosis, asthma, primary sclerosing cholangitis [8] or pancreatic cancer [78] and others that were not previously described associated with CFTR dysfunction, such as cirrhosis or intestina atresia [9]. Despite a number of limitations of this study, notably the absence of any data about *CFTR* testing (the number and kind of variants is not known, a number of CF carriers might have a CFTR-RD or bear a second *CFTR* variant), and the low absolute risk for a carrier to develop each condition, the results of the study, if confirmed, would challenge the status of “healthy carrier” and open a new era in personalized preventive medicine. This would also lead to dramatically modify the message given to the parents of a so-called “healthy” carrier detected through NBS, both for the child and the carrier parent, as well as to all carriers identified through family cascade testing or preconception carrier screening. Genetic counseling should be very cautious with such data, which should also be discussed keeping in mind the presumed heterozygote selective advantage, at least for carriers of F508del [79]. Especially in the perspective of expanded carrier screening in the general population, the risk is again to overestimate *CFTR* variants as CF-causing, hence overpredict healthy individuals at risk for developing a number of diseases. As long as the penetrance associated with *CFTR* variants is not known, implementation of genomic analysis for CF NBS and genetic counseling purposes appear detrimental. An optimal compromise would be to perform NGS with bioinformatics targeting a wide panel of fully penetrant CF-causing variants, as recently implemented for CF NBS [73,74]. It seems we are moving from a technological challenge towards a societal, political and ethical challenge.

## Figures and Tables

**Table 1:** Data available in *CFTR*-France and CFTR2 according to categories of evidence of variant pathogenicity.

Categories of Evidence	*CFTR*-France		CFTR2	
Population data: general population	+	Link to dbSNP and gnomAD	+	Reference to general population and CF carriers analysis for incomplete penetrance of *CFTR* variants
Population data: clinical observations	+	- 853 variants in about 5000 CF and CFTR-RD patients, and asymptomatic compound heterozygous individuals (data collected in molecular genetics laboratories, cross-reference with the French CF Registry) - Per patient: Age at diagnosis, symptoms, pancreatic status, meconium ileus, sweat chloride values, NBS - Link to CF Mutation Database and CFTR2	+	- 432 variants in about 89,000 CF patients (data collected from national registries) - Aggregated data for a given variant or genotype: age, lung and pancreatic function, *Pseudomonas aeruginosa* infection, sweat chloride values - Reference to ClinVar and LOVD
Literature	+	Link to PubMed for functional data	+	Link to PubMed for clinical and functional data
Computational predictions	+	AGVGD, MAPP, SIFT, PolyPhen-2, CYSMA	-	
Allelic and segregation data	+	- Data on variants identified in *trans* - Data on complex alleles	+	- Data provided on specific genotypes - No data on complex alleles
Functional data	+	Link to PubMed (transcript and protein studies)	+	- Data on CFTR protein maturation, folding, quantity and function in different cell lines - Link to PubMed

+: data available in the locus specific databases; -: data unavailable in the locus specific databases; LOVD: Leiden Open Variant Database [45]; NBS: newborn screening.

## Supplementary Materials

The following are available online at https://www.mdpi.com/2073-4425/11/6/619/s1, Table S1. Sensitivity of the ACMG recommended CF-causing variant panel for CF carrier screening in different countries and ethnic populations. ACMG: American College of Medical Genetics. Allelic frequencies among CF alleles are indicated in percentage. 0 (cells in grey) indicates that the variant was absent in the population tested. Table S2: Frequency in different countries of non-F508del variants approved for CFTR modulator therapy. * Variants approved by Food and Drug Administration (FDA) for ivacaftor in a first list and ** in the second list. Variants approved by European Medicines Agency (EMA) are in grey; £ Variants approved by FDA for tezacaftor/ivacaftor when in combination with F508del; § Variants approved by EMA for tezacaftor/ivacaftor; 0 indicates that the variant was absent in the population tested; nd*: frequency < 0.01%. The total does not reflect the approval in each country.
