# Supplementary material for: Molecular Diagnosis and Genetic Counseling of Cystic Fibrosis and Related Disorders: New Challenges
Source: Genes (Basel). 2020 Jun 4;11(6):619. doi: 10.3390/genes11060619 (PMC7349214; doi:10.3390/genes11060619)
Supplement: Supplementary file 1 [file genes-11-00619-s001.docx]

**Supplementary Materials** to the manuscript “**Molecular Diagnosis and Genetic Counseling of Cystic Fibrosis and Related Disorders: New Challenges**” by Thierry Bienvenu, Maureen Lopez and Emmanuelle Girodon

Table S1: Sensitivity of the ACMG recommended CF-causing variant panel for CF carrier screening in different countries and ethnic populations

|  | | US (pan-ethnic) [1] | Canada (Lac St Jean) [2] | United Kingdom (Wales) [2] | Belgium [2] | France [3] | Germany [2] | Italy (South) [4] | Czech Republic [5] | Slovakia [6] | Bulgarian [7] | North Macedonia [8] | Chechen Republic [9] | Turkey [10] | Western Iran [11] | Saudi Arabia [12] | China [13] | China [14] | China [15] | Brazil [16] | Ecuador [17] |
| --- | --- | --- | --- | --- | --- | --- | --- | --- | --- | --- | --- | --- | --- | --- | --- | --- | --- | --- | --- | --- | --- |
| Variants (HGVS) | Variants (legacy) |  |  |  |  |  |  |  |  |  |  |  |  |  |  |  |  |  |  |  |  |
| c.1521_1523del | F508del | 66.31 | 59 | 71.6 | 75.1 | 67.24 | 71.8 | 51.5 | 67 | 60.36 | 55 | 75.9 | 0 | 23.5 | 18.5 | 11.4 | 0 | 2.5 | 0 | 56.3 | 24.7 |
| c.1519_1522del | I507del | 0.9 | 0 | 1.1 | 0.6 | 0.63 | 0 | 0.1 | 0.08 | 0 | 0 | 0 | 0 | 0 | 0 | 0 | 0 | 0 | 0 | 0 | 0 |
| c.489+1G>T | 621+1G>T | 1.3 | 24.3 | 6.6 | 0 | 0.26 | 0 | 0 | 0.42 | 0.36 | 1.43 | 1.3 | 0 | 0.6 | 0 | 0 | 0 | 0 | 0 | 0.29 | 0.6 |
| c.254G>A | G85E | 0.26 | 0.8 | 0 | 0 | 0.34 | 0 | 0.3 | 0.17 | 0.18 | 0.36 | 0 | 0 | 0 | 0 | 0.16 | 0 | 0 | 0 | 0.59 | 11.1 |
| c.1657C>T | R553X | 1.21 | 0 | 1.1 | 0 | 1.29 | 2 | 1.5 | 0.5 | 1.45 | 0 | 0 | 0 | 0 | 0 | 0.47 | 9 | 2.5 | 2.5 | 0.29 | 0 |
| c.1624G>T | G542X | 2.64 | 0.5 | 2.2 | 3.5 | 3.19 | 1.2 | 5.9 | 2 | 4.73 | 3.93 | 3.5 | 0 | 3.6 | 0 | 0.16 | 0 | 0 | 0 | 7.62 | 2.3 |
| c.350G>A | R117H | 0.54 | 0 | 0.5 | 0 | 0.3 | 0 | 0 | 0 | 1.27 | 0 | 0 | 0 | 0 | 0 | 0 | 0 | 0 | 0 | 0 | 0 |
| c.1000C>T | R334W | 0.37 | 0 | 0 | 0 | 0.3 | 0 | 0.1 | 0.25 | 0.18 | 0.71 | 0 | 1.55 | 0 | 40.74 | 0 | 0 | 0 | 1.6 | 2.64 | 0.6 |
| c.3718-2477C>T | 3849+10kbC>T | 0.85 | 0 | 0.5 | 0 | 0.13 | 1 | 0.9 | 1.67 | 4.18 | 1.43 | 0 | 0 | 0 | 0 | 0 | 0 | 0 | 0 | 0 | 0 |
| c.3484C>T | R1162X | 0.3 | 0 | 0 | 0.5 | 0.56 | 0 | 0 | 0.33 | 0 | 0.36 | 0 | 0 | 0 | 0 | 0 | 0 | 0 | 0 | 2.64 | 0 |
| c.1652G>A | G551D | 1.93 | 0 | 2.2 | 0 | 1.16 | 0.9 | 0 | 2.92 | 0.18 | 0 | 0 | 0 | 0 | 0 | 0 | 0 | 0 | 0 | 0 | 0 |
| c.1585-1G>A | 1717-1G>A | 0.44 | 0 | 0 | 1.6 | 1.47 | 0.9 | 1.8 | 0.33 | 0.55 | 0 | 0 | 0 | 0.6 | 0 | 0 | 0 | 0 | 0 | 0.88 | 0 |
| c.1040G>C | R347P | 0.36 | 0 | 0 | 0 | 0.56 | 1.2 | 0.5 | 0.92 | 0 | 1.79 | 0.9 | 0 | 0.6 | 0 | 0 | 0 | 0 | 0 | 0.29 | 0 |
| c.2657+5G>A | 2789+5G>A | 0.38 | 0 | 0 | 0 | 1.42 | 0 | 0.7 | 0.5 | 0 | 1.79 | 0.6 | 0 | 2.4 | 0 | 0.16 | 0 | 0 | 0 | 0.59 | 0 |
| c.2052delA | 2184delA | 0.15 | 0 | 0 | 0 | 0 | 0 | 0 | 0.08 | 0 | 0.36 | 0 | 0 | 0 | 0 | 0 | 0 | 0 | 0 | 0.59 | 0 |
| c.3846G>A | W1282X | 2.2 | 0 | 0 | 1.4 | 0.52 | 0.7 | 2.2 | 0.58 | 1.27 | 1.43 | 0 | 1.55 | 3 | 0 | 0 | 0 | 0 | 0 | 0.59 | 0 |
| c.1364C>A | A455E | 0.26 | 8.2 | 0 | 1 | 0.22 | 0 | 0 | 0 | 0 | 0 | 0 | 0 | 0 | 0 | 0 | 0 | 0 | 0 | 0 | 0 |
| c.570+1G>T | 711+1G>T | 0.35 | 1 | 0 | 0 | 0.67 | 0 | 0.7 | 0.08 | 0.55 | 0 | 0.3 | 0 | 0 | 0 | 10 | 0 | 0 | 0 | 0.59 | 0 |
| c.3528delC | 3659delC | 0.28 | 0 | 0 | 0 | 0.41 | 0.6 | 0 | 0.17 | 0 | 0 | 0 | 0 | 0 | 0 | 0 | 0 | 0 | 0 | 0 | 0 |
| c.2988+1G>A | 3120+1G>A | 0.86 | 0 | 0 | 0 | 0.82 | 0 | 0 | 0 | 0.18 | 0 | 0 | 0 | 0 | 0 | 0 | 0 | 0 | 0 | 0.88 | 0 |
| c.3909C>G | N1303K | 1.27 | 0.5 | 0.5 | 2.7 | 1.98 | 1.8 | 7.3 | 2.42 | 2.55 | 4.29 | 1.9 | 0 | 2.4 | 0 | 0 | 0 | 0 | 0 | 3.23 | 4.1 |
| c.1679G>C | R560T | 0.3 | 0 | 0 | 0 | 0 | 0 | 0 | 0 | 0 | 0 | 0 | 0 | 0 | 0 | 0 | 0 | 0 | 0 | 0 | 0 |
| c.1766+1G>A | 1898+1G>A | 0.13 | 0 | 5.5 | 0 | 0.001 | 0 | 0 | 1.42 | 0.36 | 0.36 | 0 | 0 | 0 | 0 | 0 | 0 | 0 | 0 | 0 | 0 |
| Total frequency (%) | | 83.59 | 94.3 | 91.8 | 86.4 | 83.47 | 82.1 | 73.5 | 81.84 | 78.36 | 73.24 | 84.4 | 3.1 | 36.7 | 59.24 | 22.35 | 9 | 5 | 4.1 | 78.01 | 43.4 |

ACMG: American College of Medical Genetics. Allelic frequencies among CF alleles are indicated in percentage. 0 (cells in grey) indicates that the variant was absent in the population tested.

Table S2: Frequency in different countries of non-F508del variants approved for CFTR modulator therapy

|  |  | United States (42737) [1] | Belgium (1504) [2] | France (2320) [3] | Germany (1154) [18] | Italy (South) (742) [4] | Czech Republic (1200) [5] | Slovakia (550) [6] | Bulgaria (280) [7] | North Macedonia (316) [8] | Russia (208) [19] | Chenchen Republic (64) [9] | Turkey (166) [10] | West Iran (54) [11 | Saudi Arabia (792) [12] | China (44) [13] | China (122) [14] | Brazil (338) [16] | Ecuador (282) [17] |
| --- | --- | --- | --- | --- | --- | --- | --- | --- | --- | --- | --- | --- | --- | --- | --- | --- | --- | --- | --- |
| Variants (HGVS) | Variants (legacy name) |  |  |  |  |  |  |  |  |  |  |  |  |  |  |  |  |  |  |
| c.532G>A | G178R* | nd* | 0 | 0.09 | 0 | 0.3 | 0 | 0.18 | 1.07 | 0 | 0 | 0 | 0 | 0 | 0 | 0 | 0 | 0 | 0 |
| c.1652G>A | G551D* | 1.93 | 0 | 1.16 | 1.2 | 0 | 2.92 | 0.18 | 0 | 0 | 0 | 0 | 0 | 0 | 0 | 0 | 0 | 0 | 0 |
| c.1651G>A | G551S* | nd* | 0 | 0 | 0 | 0 | 0 | 0 | 0 | 0 | 0 | 0 | 0 | 0 | 0 | 0 | 0 | 0 | 0 |
| c.3731G>A | G1244E* | nd* | 0 | 0.17 | 0 | 0.8 | 0 | 0 | 0 | 0 | 0 | 0 | 0 | 0 | 0 | 0 | 0 | 0 | 0 |
| c.4046G>A | G1349D* | nd* | 0 | 0 | 0 | 0.8 | 0 | 0 | 0.36 | 1.6 | 0 | 0 | 0 | 0 | 0 | 0 | 0 | 0 | 0 |
| c.1646G>A | S549N* | 0.14 | 0 | 0 | 0 | 0 | 0 | 0 | 0 | 0 | 0 | 0 | 0 | 0 | 0 | 0 | 0 | 0 | 0 |
| c.1645A>C, c.1647T>G | S549R* | nd* | 0 | 0 | 0 | 0.1 | 0 | 0 | 0 | 0 | 0 | 0 | 0 | 0 | 3.5 | 0 | 0 | 0.59 | 1.17 |
| c.3752G>A | S1251N* | nd* | 1 | 0.3 | 0.17 | 0 | 0 | 0 | 0 | 0 | 0 | 0 | 0 | 0 | 0 | 0 | 0 | 0 | 0 |
| c.3763T>C | S1255P* | nd* | 0.5 | 0 | 0 | 0 | 0 | 0 | 0 | 0 | 0.96 | 0 | 0 | 0 | 0 | 0 | 0 | 0 | 0 |
| c.1364C>A | A455E**;£; § | 0.26 | 1 | 0.22 | 0.08 | 0 | 0 | 0 | 0 | 0 | 0.96 | 0 | 0 | 0 | 0 | 0 | 0 | 0 | 0 |
| c.1399G>A | A1067T**, £ | nd* | 0 | 0 | 0 | 0 | 0 | 0 | 0 | 0 | 0 | 0 | 0 | 0 | 0 | 0 | 0 | 0 | 0 |
| c.330C>A | D110E**, £ | nd* | 0 | 0 | 0 | 0 | 0 | 0 | 0 | 0 | 0 | 0 | 0 | 0 | 0 | 0 | 0 | 0 | 0 |
| c.328G>C | D110H**, £ | nd* | 0 | 0 | 0 | 0.1 | 0.17 | 0 | 0 | 0 | 0 | 0 | 0.6 | 2 | 0 | 0 | 0 | 0.29 | 0 |
| c.1736A>G | D579G**, £, § | nd* | 0 | 0 | 0 | 0.8 | 0 | 0 | 0 | 0 | 0 | 0 | 0 | 0 | 0 | 0 | 0 | 0 | 0 |
| c.3454G>C | D1152H**, £, § | 0.03 | 0 | 0 | 0 | 0.3 | 0.33 | 0.36 | 0 | 0 | 0 | 0 | 0 | 0 | 0 | 0 | 0 | 0.29 | 0 |
| c.3808G>A | D1270N**,£ | 0.04 | 0 | 0 | 0 | 0 | 0 | 0 | 0 | 0 | 0 | 0 | 0 | 0 | 0 | 0 | 0 | 0.29 | 0 |
| c.166G>A | E56K**, £ | nd* | 0 | 0 | 0 | 0 | 0 | 0 | 0 | 0 | 0 | 0 | 0 | 0 | 0 | 0 | 0 | 0 | 0 |
| c.577G>A | E193K**,£ | nd* | 0 | 0 | 0 | 0 | 0 | 0 | 0 | 0 | 0 | 0 | 0 | 0 | 0 | 0 | 0 | 0 | 0 |
| c.3154T>G | F1052V**, £ | nd* | 0 | 0 | 0 | 0 | 0 | 0.18 | 0 | 0 | 0 | 0 | 3 | 0 | 0 | 0 | 0 | 0 | 0 |
| c.3222T>A | F1074L**, £ | nd* | 0 | 0 | 0 | 0 | 0 | 0 | 0 | 0 | 0 | 0 | 0 | 0 | 0 | 0 | 0 | 0 | 0 |
| c.3205G>A | G1069R** | nd* | 0 | 0 | 0 | 0 | 0 | 0.18 | 0.36 | 0 | 0 | 0 | 0 | 0 | 0 | 0 | 0 | 0 | 0 |
| c.3179A>C | K1060T**, £ | nd* | 0 | 0 | 0 | 0 | 0 | 0 | 0 | 0 | 0 | 0 | 0 | 0 | 0 | 0 | 0 | 0 | 0 |
| c.617T>G | L206W**, £, § | nd* | 0 | 0.34 | 0 | 0 | 0 | 0 | 0 | 0 | 0 | 0 | 0 | 0 | 0 | 0 | 0 | 0.29 | 0 |
| c.200C>T | P67L**, £, § | nd* | 0 | 0 | 0 | 0 | 0 | 0 | 0 | 0 | 0 | 0 | 0 | 0 | 0 | 0 | 0 | 0 | 0 |
| c.220C>T | R74W**, £ | nd* | 0 | 0 | 0 | 0 | 0 | 0 | 0 | 0 | 0 | 0 | 0.6 | 0 | 0 | 0 | 0 | 0.29 | 0 |
| c.4349C>T | R117C**, £, § | nd* | 0 | 0.09 | 0.08 | 0 | 0.08 | 0 | 0 | 0.6 | 1.92 | 0 | 0 | 0 | 0 | 0 | 0 | 0 | 0 |
| c.1040G>A | R347H**, £ | 0.06 | 0 | 0.04 | 0 | 0 | 0.08 | 0 | 0 | 0 | 0 | 0 | 0 | 0 | 0 | 0 | 0 | 0 | 0 |
| c.1055G>A | R352Q**, £, § | nd* | 0 | 0.13 | 0 | 0 | 0 | 0 | 0 | 0 | 0 | 0 | 0 | 0 | 0 | 0 | 0 | 0 | 0 |
| c.3209G>A | R1070Q**, £ | nd* | 0 | 0 | 0 | 0 | 0 | 0.18 | 0.71 | 0 | 0 | 0 | 1.8 | 0 | 0 | 0 | 0 | 0.29 | 0 |
| c.3298C>T | R1070W**, £, § | nd* | 0 | 0 | 0 | 0 | 0 | 0 | 0 | 0 | 0 | 0 | 0 | 0 | 0 | 0 | 0 | 0 | 0 |
| c.2834C>T | S945L**, £, § | nd* | 0 | 0.04 | 0 | 0 | 0.5 | 0 | 0 | 0 | 1.92 | 0 | 0 | 0 | 0 | 0 | 0 | 0 | 0 |
| c.2930C>T | S977F**, £, § | nd* | 0 | 0 | 0 | 0 | 0 | 0 | 0 | 0 | 0 | 0 | 0 | 0 | 0 | 0 | 0 | 0 | 0 |
| c.579+3A>G | 711+3A>G**, £, § | nd* | 0 | 0 | 0 | 0 | 0.17 | 0 | 0.36 | 0 | 0 | 0 | 0 | 0 | 0 | 0 | 0 | 0 | 0 |
| c.2657+5G>A | 2789+5G>A**, £, § | 0.38 | 0 | 1.42 | 0.86 | 0.7 | 0.5 | 0 | 1.79 | 0.6 | 0 | 0 | 0 | 0 | 0 | 0 | 0 | 0.59 | 0 |
| c.350G>A | R117H** | 0.54 | 0 | 0.3 | 0.26 | 0 | 0 | 1.27 | 0 | 0 | 0 | 0 | 0 | 0 | 0 | 0 | 0 | 0 | 0 |
| c.3140-26A>G | 3272-26A>G,£, § | nd* | 1 | 0.65 | 0.95 | 0.1 | 0.67 | 1.27 | 0 | 0 | 0 | 0 | 0 | 0 | 0 | 0 | 0 | 0.59 | 0 |
| c.3718-2477C>T | 3849+10kbC>T £, § | nd* | 0 | 0.13 | 1.21 | 0.9 | 1.67 | 4.18 | 1.43 | 0 | 0 | 0 | 0 | 0 | 0 | 0 | 0 | 0 | 0 |
| c.2491G>T | E831*, £ | nd* | 0 | 0.09 | 0 | 0 | 0 | 0.18 | 0.71 | 0 | 0 | 0 | 0 | 0 | 0 | 0 | 0 | 0 | 0 |
| Total (%) | | 3.38 | 3.5 | 5.17 | 4.81 | 4.9 | 7.09 | 8.16 | 6.79 | 2.8 | 5.76 | 0 | 6 | 2 | 3.5 | 0 | 0 | 3.51 | 1.17 |

* Variants approved by Food and Drug Administration (FDA) for ivacaftor in a first list and ** in the second list. Variants approved by European Medicines Agency (EMA) are in grey; £ Variants approved by FDA for tezacaftor/ivacaftor when in combination with F508del; § Variants approved by EMA for tezacaftor/ivacaftor; 0 indicates that the variant was absent in the population tested; nd*: frequency <0.01%. The total does not reflect the approval in each country.

References

1. Watson, M.S.; Cutting, G.R.; Desnick, R.J.; Driscoll, D.A.; Klinger, K.; Mennuti, M.; Palomaki, G.E.; Popovich, B.W.; Pratt, V.M.; Rohlfs, E.M.; et al. Cystic fibrosis population carrier screening: 2004 revision of American College of Medical Genetics mutation panel. Genet. Med. 2004, 6, 387–391, doi:10.1097/01.GIM.0000139506.11694.7C.

2. Bobadilla, J.L.; Macek, M.; Fine, J.P.; Farrell, P.M. Cystic fibrosis: A worldwide analysis of CFTR mutations-correlation with incidence data and application to screening. Hum. Mutat. 2002, 19, 575–606, doi:10.1002/humu.10041.

3. Audrézet, M.P.; Munck, A.; Scotet, V.; Claustres, M.; Roussey, M.; Delmas, D.; Férec, C.; Desgeorges, M. Comprehensive CFTR gene analysis of the French cystic fibrosis screened newborn cohort: Implications for diagnosis, genetic counseling, and mutation-specific therapy. Genet. Med. 2015, 17, 108–116, doi:10.1038/gim.2014.113.

4. Castaldo, G.; Polizzi, A.; Tomaiuolo, R.; Cazeneuve, C.; Girodon, E.; Santostasi, T.; Salvatore, D.; Raia, V.; Rigillo, N.; Goossens, M.; et al. Comprehensive Cystic Fibrosis mutation epidemiology and haplotype characterization in a Southern Italian population. Ann. Hum. Genet. 2005, 69, 15–24, doi:10.1046/j.1529-8817.2004.00130.x.

5. Křenková, P.; Piskáčková, T.; Holubová, A.; Balaščaková, M.; Krulišová, V.; Čamajová, J.; Turnovec, M.; Libik, M.; Norambuena, P.; Štambergová, A.; et al. Distribution of CFTR mutations in the Czech population: Positive impact of integrated clinical and laboratory expertise, detection of novel/de novo alleles and relevance for related/derived populations. J. Cyst. Fibros. 2013, 12, 532–537, doi:10.1016/j.jcf.2012.12.002.

6. Soltysova, A.; Tothova Tarova, E.; Ficek, A.; Baldovic, M.; Polakova, H.; Kayserova, H.; Kadasi, L. Comprehensive genetic study of cystic fibrosis in Slovak patients in 25 years of genetic diagnostics. Clin. Respir. J. 2018, 12, 1197–1206, doi:10.1111/crj.12651.

7. Petrova, G.; Yaneva, N.; Hrbková, J.; Libik, M.; Savov, A.; Macek, M. Identification of 99% of CFTR gene mutations in Bulgarian-, Bulgarian Turk-, and Roma cystic fibrosis patients. Mol. Genet. Genom. Med. 2019, 7, e696, doi:10.1002/mgg3.696.

8. Terzic, M.; Jakimovska, M.; Fustik, S.; Jakovska, T.; Sukarova-Stefanovska, E.; Plaseska-Karanfilska, D. Cystic fibrosis mutation spectrum in north Macedonia: A step toward personalized therapy. Balk. J. Med. Genet. 2019, 22, 35–40, doi:10.2478/bjmg-2019-0009.

9. Petrova, N.V.; Kashirskaya, N.Y.; Saydaeva, D.K.; Polyakov, A.V.; Adyan, T.A.; Simonova, O.I.; Gorinova, Y.V.; Kondratyeva, E.I.; Sherman, V.D.; Novoselova, O.G.; et al. Spectrum of CFTR mutations in Chechen cystic fibrosis patients: High frequency of c.1545_1546delTA (p.Tyr515X;1677delTA) and c.274G > A (p.Glu92Lys, E92K) mutations in North Caucasus. BMC Med. Genet. 2019, 20, 44, doi:10.1186/s12881-019-0785-z.

10. Kılınç, M.O.; Ninis, V.N.; Dağlı, E.; Demirkol, M.; Özkınay, F.; Arıkan, Z.; Çoğulu, Ö.; Hüner, G.; Karakoç, F.; Tolun, A. Highest heterogeneity for cystic fibrosis: 36 mutations account for 75% of all CF chromosomes in Turkish patients. Am. J. Med. Genet. 2002, 113, 250–257, doi:10.1002/ajmg.10721.

11. Karimi, N.; Alibakhshi, R.; Almasi, S. CFTR Mutation Analysis in Western Iran: Identification of Two Novel Mutations. J. Reprod. Infertil. 2018, 19, 3–9.

12. Banjar, H.; Al-Mogarri, I.; Nizami, I.; Al-Haider, S.; AlMaghamsi, T.; Alkaf, S.; Al-Enazi, A.; Moghrabi, N. Geographic distribution of cystic fibrosis transmembrane conductance regulator (CFTR) gene mutations in Saudi Arabia. Int. J. Pediatric Adolesc. Med. 2019, doi:10.1016/j.ijpam.2019.12.002.

13. Zheng, B.; Cao, L. Differences in gene mutations between Chinese and Caucasian cystic fibrosis patients. Pediatric Pulmonol. 2017, 52, E11–E14, doi:10.1002/ppul.23539.

14. Tian, X.; Liu, Y.; Yang, J.; Wang, H.; Liu, T.; Xu, W.; Li, X.; Zhu, Y.; Xu, K.-F.; Zhang, X. p.G970D is the most frequent CFTR mutation in Chinese patients with cystic fibrosis. Hum. Genome Var. 2016, 3, 15063, doi:10.1038/hgv.2015.63.

15. Guo, X.; Liu, K.; Liu, Y.; Situ, Y.; Tian, X.; Xu, K.-F.; Zhang, X. Clinical and genetic characteristics of cystic fibrosis in Chinese patients: A systemic review of reported cases. Orphanet J. Rare Dis. 2018, 13, 224, doi:10.1186/s13023-018-0968-2.

16. Pereira, S.V.-N.; Ribeiro, J.D.; Ribeiro, A.F.; Bertuzzo, C.S.; Marson, F.A.L. Novel, rare and common pathogenic variants in the CFTR gene screened by high-throughput sequencing technology and predicted by in silico tools. Sci. Rep. 2019, 9, 6234, doi:10.1038/s41598-019-42404-6.

17. Ruiz-Cabezas, J.C.; Barros, F.; Sobrino, B.; García, G.; Burgos, R.; Farhat, C.; Castro, A.; Muñoz, L.; Zambrano, A.K.; Martínez, M.; et al. Mutational analysis of CFTR in the Ecuadorian population using next-generation sequencing. Gene 2019, 696, 28–32, doi:10.1016/j.gene.2019.02.015.

18. The Molecular Genetic Epidemiology of Cystic Fibrosis: Report of a Joint Meeting of WHO/ECFTN/ICF(M)A/ECFS, Genoa, Italy, 19 June 2002. Available online: https://apps.who.int/iris/handle/10665/68702 (accessed on 7 May 2020).

19. Petrova, N.V.; Marakhonov, A.V.; Vasilyeva, T.A.; Kashirskaya, N.Y.; Ginter, E.K.; Kutsev, S.I.; Zinchenko, R.A. Comprehensive genotyping reveals novel CFTR variants in cystic fibrosis patients from the Russian Federation. Clin. Genet. 2019, 95, 444–447, doi:10.1111/cge.13477.
